# Water-Drinking and Drinking-Water

**Published:** 1912-06

**Authors:** 

**Affiliations:** Eisenach, Doctor of Physiology Translated Extract from the Balneologische Zeitung (Berlin) of the 20th January, 1912, No. 2


### ABSTRACTS

### Water-Drinking and Drinking-Water

By DR. KUHNER, of Eisenach, Doctor of Physiology
Translated Extract from the Balneologische Zeitung (Berlin) of the 20th January, 1912, No. 2.

THE question as to the use of drinking water at meals is still in dispute, many doctors holding to the teachings of the Salernitan School that the juices of the stomach may become too dilute, so that digestion is impeded.

This is an error. Since the ferments have been studied and found to be of enormous strength, especially so with the diastases, the assumption appears to me to be untenable that water drinking during meals could hinder the process of digestion. Also the free hydrochloric acid contained in the juices of the stomach might be diluted to a far greater extent than is effected through drinking water at meals without the digestion suffering in the least. The effect of ferments in the stomach is rapidly produced even by solutions, which are only weakly acidulated. Moreover, the Russian Investigator—Pawlow—found that immediately after thorough washing of the stomach, the degree of acidity is unaltered. Finally Ruzika, by exact experiments on himself, showed that half a litre of water drunk during the midday meal did not hinder the absorption from nutritive substances at all. On the contrary, if one reduces the fluidity of the food too greatly there is incomplete contact between the ferments and the hydrochloric acid on the one hand, and the food on the other, and the solution by the pepsin is seriously delayed. Then again, if we keep to a dry diet too long, the effect on the albuminoids is left incomplete. The individual suffers seriously from loss of nourishment, fat and muscles are greatly reduced and the person rapidly grows thinner. . . . The quality of the drinking water is no less important than the quantity. What are the requirements which one should lay down for the quality of the water? In the first place by bacteriologic examination it must be ascertained that the water is free from pathogenic microbes. Even the colon bacilli which are present in the drinking water of many places in large quantities are not above suspicion. Fortunately, consumers are able, by boiling for 40 minutes, to sterilise any water sufficiently to make it potable. Any drinking water should be rejected which contains more than 100 germs in a cubic centimetre.

. . . Other surface waters too are very frequently tainted. The increasing overpopulation of Germany and its manufacturing industries work in this direction, the flushing drainage system

(without filtering-beds) being especially responsible for the pollution of the rivers. It is very regrettable that so many parishes allow the solids simply to pass into the river instead of by a rational system employing same for agricultural purposes. In his treatise on "The Necessity for Keeping German Waters Clean" Bonne complains as to this: "The Germans," he says, "have a high degree of culture, but at the same time stand on the lowest step of uncivilization. While they have dirigible airships and light and heat and motive power from electricity along with the telegraph and telephone, they still allow the whole of their waste to run into their rivers and lakes, instead of using it to cultivate their heaths and stretches of sand. Fish die in the poisoned water. But human beings wash in and drink it, although, even diluted and filtered several times it still contains impurities of human origin and often is the cause of great epidemics of disease."

This Jeremiad is not without reason. How far are we from the hygienic principles of the Persians of whom Herodotus tells, that they reverenced the rivers and would not allow people to wash their hands in them or any impurities to be thrown into them. Today we smile at such devout simplicity, but we cease to do so when from our polluted rivers vengeful plagues arise, as in 1892 cholera from the waters of the Elbe at Hamburg, and in 1901, typhoid at Gelsenkirchen from the river Ruhr. The first act of the tragedy is always pollution, the second, disease, for it is in the dirt that bacteria flourish. What is so called self-purification of the rivers is only apparent, and is no real cleansing; it is a hypothesis to deprecate sins against hygiene, and it calms our scruples. In reality the impurities which are conducted into the rivers are only hidden; they go out of sight, but they have not lost their power for harm. The apparent self-cleansing of a river chiefly means that down the stream far from the place where the impurities have entered, and after much mud has sunk to the bottom of the river, and especially after this river has taken up the waters of many tributaries, which may still be clean, the water of the river becomes purer again. But there can really be no talk of any actual self-cleansing of a river.

